# Microbial and metabolomic insights into the bovine lipometabolic responses of rumen and mammary gland to zymolytic small peptide supplementation

**DOI:** 10.3389/fvets.2022.875741

**Published:** 2022-09-14

**Authors:** En Liu, Weiwei Xiao, Qijian Pu, Lanjiao Xu, Long Wang, Kang Mao, Wei Hong, Mingren Qu, Fuguang Xue

**Affiliations:** ^1^Jiangxi Province Key Laboratory of Animal Nutrition/Engineering Research Center of Feed Development, Jiangxi Agricultural University, Nanchang, China; ^2^Chengdu Mytech Biotech Co., Ltd., Chengdu, China; ^3^Shanghai Menon Animal Nutrition Technology Co., Ltd., Shanghai, China; ^4^Nanchang Key Laboratory of Animal Health and Safety Production, Jiangxi Agricultural University, Nanchang, China

**Keywords:** small peptide, milk fat, lipometabolic, rumen microbiota, metabolomics

## Abstract

Small peptides provide the easily utilized nitrogen for rumen microbial and promote acetate generation for milk fat synthesis. However, the impacts of peptide supplements on lipometabolic processes were still unclear. Therefore, a total of 800 multiparous dairy herds (with an average live weight of 667.6 ± 39.4 kg, an average lactation of 89.3 ± 18.8 days, and an average calving parity of 2.76 ± 0.47) were randomly allocated to the control (CON) and the small peptide (SP) supplement (100 g/day for each cow) treatments, respectively. A 35-day-long feeding procedure that includes a 7-day-long pretreatment test and a 28-day-long treatment test was followed for all cows. Dry matter intake (DMI) was recorded every day and calculated by the deviation between the supply and residue, while the daily milk production was automatically recorded through the rotary milking facilities. Milk samples were collected from each replicate on the last day, followed by the milk quality and milk lipid composition measurement. Rumen fluid samples were collected on the last day through esophageal tubing 3 h after morning feeding for the determination of the underlying mechanism of the small peptide on lipid metabolism through the measurement of rumen lipometabolic-related metabolites and rumen bacterial communities. Results indicated that dry matter intake showed an increasing trend, while milk production and the milk fat content remarkably increased after SP supplement (*P* < 0.05). Further detailed detection showed the mainly increased milk composition focused on monounsaturated fatty acid (MUFA) and polyunsaturated fatty acid (PUFA). Acetate-producing microbes, such as *Acetitomaculum, Bifidobacterium, Succiniclasticum*, and *Succinivibrio*, and butyrate-producing microbes, such as *Shuttleworthia* and *Saccharofermentans*, significantly proliferated, which causatively brought the increased ruminal content of acetate, isobutyrate, and butyrate after SP supplement (*P* < 0.05) compared with CON. Lipometabolic metabolites such as phosphatidylcholine (PC), phosphatidylethanolamine (PE), phosphatidylinositol (PI), phosphatidylserine (PS), triacylglycerol (TG), and Acetyl-CoA also significantly increased after SP supplement. In summary, SP supplements help to increase milk fat content through the proliferation of rumen bacterial communities, which provided more acetate and butyrate for milk fat synthesis combined with the promotion of ruminal lipometabolism.

## Introduction

Ruminants provide a readily available source of lipids for humans in the form of both tissue and milk fats. As one of the major assessment indices of milk quality, milk fat regulated not only the flavor but also the nutritional value of milk (1). Conventionally, studies considered that the *de novo* synthesis of milk fat is through utilizing acetate and beta-hydroxybutyric acid (BHBA) as the substrates (2–4), which are not only the main substrates for the synthesis of fatty acid (FA) in ruminants but also the significant positive regulators for the synthesis in milk fat (5). Therefore, investigating the nutritional strategies to increase rumen acetate uptake is prevalent to increase milk fat content.

The rumen is the basal nutrition supplying organ in cows to maintain the normal physiological functions by the large homeostasis microbial ecosystem that equilibrated the utilization of carbon and nitrogen sources (6). The rumen energy and nitrogen balance (RENB) provides the primary proliferation circumstances for the rumen microbiome and promotes more easily absorbable nutrients (7). Traditionally, a higher proportion of diet forage was more adapted to RENB, triggered the milk fat synthesis process, and increased the milk fat content compared with higher concentrate diets (8). However, in the modern dairy industry, energy-abundant concentrates were more preferentially offered to the lactating dairy cows to maintain high milk production, which further requires more degradable nitrogen to balance the energy. Despite the application of soybean meal and other protein-abundant materials, the provision of rumen degradable protein (RDP) is still inferior to the requirement to balance the needs of energy (9, 10). The undersupply of RDP may further suppress the proliferation of both cellulolytic and amylolytic bacteria, thus causatively inducing the decrease of acetate and further suppressing the accumulation of milk fat (11, 12). Moreover, the RENB would be disturbed, which in turn induces the recessionary of energy utilization and transportation and finally impacts the milk fat synthesis.

Demeyer and Fievez (13) suggested that low concentrations of rumen-degradable proteins potentially limit microbial growth when rations are rich in starch. Adding non-protein nitrogen (NPN) such as urea is perhaps the most financial way to form microbial protein; however, the supplement quantity should be carefully limited (14). Adding peptides helps to reestablish the RENB in the high-concentrate feeding process and also promotes the growth of both cellulolytic and amylolytic bacteria (15). Peptides could be directly used by certain bacteria for microbial protein synthesis or further degraded by peptidases into AA, which can be incorporated into microbial protein or further deaminated to VFA, CO_2_, and ammonia (9). Moreover, many species of protozoa and bacteria such as *P. ruminicola* have dipeptidase activity against a wide range of dipeptides, and intriguingly, dipeptidyl-X substrates were much more rapidly degraded than amino acyl-X substrates in certain of ruminal bacteria (16–18).

Therefore, in this study, SPs derived from enzymolytic proteins were supplemented to detect the milk fat content of high-yielding dairy cows. We hypothesized that SP supplements help to increase the milk fat content through the proliferation of rumen bacterial communities and the enhancement of ruminal lipometabolism to provide more substrates, including acetate and butyrate, for the synthesis of mammary milk fat.

## Materials and methods

Animal care and procedures followed The Chinese Guidelines for Animal Welfare, which was approved by the Animal Care and Use Committee of Jiangxi Agricultural University, with the approval number JXAULL-20210201.

### Experimental design

The experiment was conducted in the Bengbu Dairy Farm, Modern Farming (Wuhe) Co. Ltd, Anhui Province, China (32.92 N, 117.38 E). A total of 800 multiparous dairy herds with an average live weight of 667.6 ± 39.4 kg, average lactation of 89.3 ± 18.8 days, average daily milk production of 47.1 ± 1.4 kg, and average calving parity of 2.76 ± 0.47 were randomly allocated to the control treatment (CON) and the SP supplement treatment for a 35-day-long feeding procedure that includes a 7-day-long pretreatment test and a 28-day-long treatment test. Cows were distributed in an individual barn, which consisted of two columns and received the transverse ventilation system in which the wind intensity could be regulated by automatic ventilators. The barn was about 348 m long and 90 m wide, the THI index was controlled to lower than 70 throughout the year, and the lighting was 20 h light with 4 h dark. The bedding was filled with sawdust. Each column has four pens, two pens treated with CON and two pens treated with SP. Each pen contains 100 cows, which was considered as a replicate.

All cows were provided the same diets, which were formulated according to NRC (2001) to meet or exceed the energy requirement estimates of Holstein dairy cows yielding 40 kg of milk/day with 3.5% milk fat and 3.0% true protein. Diets were fed three times per day at 06:00, 13:00, and 21:00 h. Details of ingredient analysis and chemical composition of diets are shown in Table 1.

**Table 1:** Ingredients and chemical composition of the TMR (dry matter basis).

**Items**	**Content**
Ingredients (%) Corn silage	24.5
Ground corn	15.7
Cottonseed meal	3.4
Alfalfa hay	14.5
Chinese wildrye	10.2
Distillers dried grains with solubles (DDGS)	3.1
Steam-flaked corn	8.5
Soybean meal	12.0
Beet pulp	4.5
Premix^1^	3.0
NaCl	0.6
Chemical composition (%)	
DM	51.2
NE (MJ/kg)	7.13
EE	4.56
CP	17.1
RDP^2^	11.14
RDP^3^	10.76
ADF	18.6
NDF	31.7
Starch	28.9
Ca	0.69
P	0.44

1 The components contained in the premix are as follows: Fe, 1,400 mg; Cu, 1,200 mg; Mn, 2,400 mg; Zn, 5,500 mg; Se, 40 mg; Co, 30 mg; I, 90 mg, VA, 900,000 IU; VD, 700,000 IU; VE, 9,000 IU.

2 RDP with small peptide supplement, calculated by the equation of RDP = A+B[Kd/(Kd+Kp)]. A, non-protein nitrogen and soluble proteins; B, potentially degradable proteins; Kd, rumen digestibility of B; Kp, velocity of circulation in rumen.

3 RDP without small peptide supplement, calculated by the equation of RDP = A+B[Kd/(Kd+Kp)]. A, non-protein nitrogen and soluble proteins; B, potentially degradable proteins; Kd, rumen digestibility of B; Kp, velocity of circulation in rumen.

#### Small peptide preparation and offering

The small peptides used in this study were provided by the Chengdu Mytech Biotech Co., Ltd, China, which were produced through enzymatic hydrolysis of cottonseed protein combined with dephenolization. The hydrolysate SPs were categorized into four fractions based on their molecular weights, namely, <1000, 1000–2000, 2000–5000, and >5000 Da, and accounted for 68.4, 16.7, 8.3, and 5.6% of the total peptides, respectively. The RDP proportion of SP was calculated as about 94.72% of total protein content based on the following equation:


$$RDP=A+B\left[\frac{Kd}{Kd+Kp}\right]$$

Where A, non-protein nitrogens and soluble proteins; B, potentially degradable proteins; Kd, rumen digestibility of B; and Kp, velocity of circulation in rumen.

Small peptides were supplemented with 10 g/day per cow through mixing with the preparation of concentrates, and the proportion of additional percentage of peptides is about 0.39% of DMI per day.

#### Production performances measurement

The average daily intake was shown as the dry matter intake (DMI) of each treatment, which was calculated through the deviation between the supply and residue. DMI was calculated and displayed as the average of four replicates. Cows were milked three times (08:00, 14:00, and 20:00, respectively) per day, and the daily milk production was automatically recorded through the rotary milking facilities (9JRP-50P2100, Delaval, Israel).

#### Milk quality and milk lipid composition measurement

Milk samples were collected in 100-ml vials from each replicate on the last day and subsequently stored at 4°C adding 2-bromo-2-nitropropane-1,3-diol for further milk quality analysis including milk protein, milk fat, and somatic cell count.

Milk lipid composition was further analyzed according to the acetyl chloride-methanol methyl esterification method presented by Wang et al. (19). Parameters including saturated fatty acid (SFA), monounsaturated fatty acid (MUFA), and polyunsaturated fatty acid (PUFA) were measured. Simply stated, two cows of each replicate and a total of 16 samples were chosen, followed by 0.5 ml of FA triglycerides mixed thoroughly with 4.5 ml of toluene as a standard solution, followed by the chromatographic analysis using the gas chromatograph (Agilent 8860 GC, CA, USA). Parameters of chromatographic measurement were set as follows. Dicyanopropyl polysiloxane column (100 m × 0.25 mm, 0.20 mm) was applied with the temperature of the column oven as 140°C for 5 min and then gradiently increased to 240°C at 4°C/min. The injector and detector temperatures were set as 260°C and 280°C, respectively. The chromatographic peak area was obtained by the integral method and further used for quantification. The retention time was determined by the positive and negative changes of the first derivative values (20).

#### Ruminal fermentation parameters measurement

The rumen fluid samples of 16 cows (two cows in each replicate, eight cows per treatment, second lactation, similar body weight) were collected on the last day through esophageal tubing 3 h after morning feeding. The first two tubes of rumen fluid that might be mixed with saliva were removed and the pure rumen fluid was then collected to ensure the rumen microbial purity. All samples were divided into two portions. One was conducted to analyze ruminal pH, rumen volatile fatty acids (VFAs), and ammonia-N (NH3-N). The other portion was frozen in the liquid nitrogen immediately and then stored at −80°C for further microbiota and metabolites measurement. A portable type pH meter (Testo 205, Testo AG, Lenzkirch, Germany) was used for the measurement of ruminal pH immediately after the rumen fluid sample was collected. Individual and total VFAs (TVFA) in the aliquots were measured using a gas chromatograph (GC-2010, Shimadzu, Kyoto, Japan). The concentration of NH_3_-N was determined by the indophenol method, and the absorbance value was measured through UV-2600 ultraviolet spectrophotometer (Tianmei Ltd., China) at 700 nm wavelength (21).

#### Rumen microbial community measurement

Rumen fluid DNA was first extracted using the Bacterial Genome DNA Extraction Kit (DP302, TIANGEN, TIANGEN BIOTECH (BEIJING) Co., Ltd). Furthermore, the 16S rRNA gene V4 region was amplified using the universal primers 520F and 802R (F: GTGCCAGCMGCCGCGGTAA and R: GGACTACHVGGGTWTCTAAT). All PCRs were carried out with the Phusion High-Fidelity PCR Master Mix (New England Biolabs). Qiagen Gel Extraction Kit (Qiagen, Hilden, Germany) was used to purify the mixture of PCR products, followed by the generation of sequencing libraries using TruSeq^®^ DNA PCR-Free Sample Preparation Kit (Illumina, USA). The Qubit@ 2.0 Fluorometer (Thermo Scientific) and Agilent Bioanalyzer 2100 system were then applied for the assessment of the library quality, and finally, Illumina HiSeq 4000 platform (Illumina Inc., San Diego, USA) was used for the sequencing process. Quality filtering of raw tags was performed under specific filtering conditions to obtain high-quality clean tags according to the Quantitative Insights Into Microbial Ecology (QIIME, V1.7.0) quality controlling process. Sequences with similarity >97% were assigned to the same operational taxonomic unit (OTU).

#### Rumen metabolites measurement

Rumen metabolites alteration in this study was measured using LC/MS analyses method, which contains metabolites extraction, UHPLC-MS/MS analysis, data processing, and annotation.

First, 100 μl of rumen fluid was accurately quantified and transferred into 400 μl of 80% methanol solution with 0.02 mg/ml L-2-chlorophenylalanin as internal standard. The mixture was allowed to settle at −10°C and treated by high-throughput tissue crusher Wonbio-96c (Shanghai Wanbo Biotechnology Co., LTD) at 50 Hz for 6 min. Then, ultrasound was performed for all samples at 40 kHz for 30 min at 5°C and carefully transferred to the sample vials for LC-MS/MS analysis after centrifugation at 13,000 g at 4°C for 15 min.

Chromatographic separation of the metabolites was performed on a Thermo UHPLC system equipped with an ACQUITY UPLC HSS T3 (100 mm × 2.1 mm i.d., 1.8 μm; Waters, Milford, USA). The sample injection volume was 2 μl, and the flow rate was set to 0.4 ml/min. The column temperature was maintained at 40°C. During the period of analysis, all these samples were stored at 4°C.

The mass spectrometric data were collected using a Thermo UHPLC-Q Exactive HF-X Mass Spectrometer equipped with an electrospray ionization (ESI) source operating in either positive or negative ion mode. The optimal conditions were set as follows: heater temperature, 425°C; capillary temperature, 325°C; sheath gas flow rate, 50 arb; Aux gas flow rate, 13 arb; ion-spray voltage floating (ISVF), −3,500 V in negative mode and 3,500 V in positive mode; and normalized collision energy, 20–40–60 V rolling for MS/MS. Full MS resolution was 60,000, and MS/MS resolution was 7,500. Data acquisition was performed with the data dependent acquisition (DDA) mode. The detection was carried out over a mass range of 70–1,050 m/z.

After UPLC-MS analyses, the raw data were imported into the Progenesis QI 2.3 (Non-linear Dynamics, Waters, USA) for peak detection and alignment. The preprocessing results generated a data matrix that consisted of the retention time (RT), mass-to-charge ratio (m/z) values, and peak intensity.

### Statistical analysis

A normal distribution test was first conducted for production performances, ruminal pH, and ruminal fermentation variables using the SAS procedure “proc univariate data = test normal,” and subsequently, the one-way ANOVA S-N-K test of SAS was performed (SAS Institute, Inc., Cary, NC, USA). Significance would be considered when *P* < 0.05, while a tendency was considered when 0.05 ≤ *P* < 0.10. OTU abundances of each rumen bacteria were first conducted a percentage transformation, and then, the one-way ANOVA S-N-K test of SAS 9.2 was performed for the differential analysis. Alpha diversity and beta diversity in our samples were calculated with QIIME 2 (22) and displayed with R software (Version 3.3.1, R Core Team, Vienna, Austria). Principal coordinate analysis (PCoA) for different rumen methanogens was conducted using R “vegan package.”

For metabolomic analysis, a multivariate statistical analysis was performed using ropls (Version1.6.2, http://bioconductor.org/packages/release/bioc/html/ropls.html) R package from Bioconductor on Majorbio Cloud Platform (https://cloud.majorbio.com). Principle component analysis (PCA) using an unsupervised method was performed to obtain an overview of the metabolic data, general clustering, trends, and outliers. All of the metabolite variables were scaled to unit-variances prior to conducting the PCA. Orthogonal partial least squares discriminate analysis (OPLS-DA) was used for statistical analysis to determine global metabolic changes between comparable groups. All of the metabolite variables were scaled to Pareto scaling prior to conducting the OPLS-DA. Variable importance in the projection (VIP) was calculated in OPLS-DA model. *P*-values were estimated with paired Student's *t*-test on single dimensional statistical analysis.

## Results

### Production performances, milk quality, and milk fat composition

The production performances included dry matter intake (DMI), average daily milk production (AMP), and the fat correlated milk (FCM); the milk quality parameters included milk fat, milk protein, milk dry matter content, colony forming unit (CFU), and somatic cell counts; and the results are shown in Table 2. The daily milk production of each cow is shown in Supplementary Table 1.

**Table 2:** Effects of small peptide supplement on the production performances (*n* = 4) and milk quality (*n* = 800).

**Items**	**SP**	**CON**	***P*-value**
DMI^1^ (kg/d)	25.86 ± 0.85	25.58 ± 0.97	0.231
DMI^2^ (kg/d)	26.55 ± 0.98	25.76 ± 1.25	0.076
AMP^1^ (kg/d)	47.62 ± 0.64	47.21 ± 0.66	0.307
AMP^2^ (kg/d)	48.41 ± 0.67	47.52 ± 0.54	0.044
FCM (kg/d)	46.59 ± 0.63	44.74 ± 0.43	0.013
Milk fat (%)	3.75 ± 0.12	3.61 ± 0.08	0.012
Milk protein (%)	3.42 ± 0.02	3.39 ± 0.03	0.107
Milk Dry matter content (%)	12.72 ± 0.13	12.53 ± 0.11	0.061
CFU(×10^3^ /mL)	2.40 ± 0.23	2.33 ± 0.13	0.722
SCC(×10^3^ /mL)	8.80 ± 0.64	9.20 ± 0.65	0.572

SP, Small peptide supplement treatment; CON, control treatment; DMI^1^, dry matter intake before treatment; DMI^2^, dry matter intake after treatment; AMP^1^, average milk production before treatment; AMP^2^, average milk production after treatment. FCM, fat-correlated milk (kg/day) = 0.4× milk yield (kg/day) + 15× milk yield (kg/day) × fat (%). CFU, colony forming unit; SCC, somatic cell counts.

The DMI and AMP were measured before SP supplement and after receiving SP supplement treatment. Based on the results, the dry matter intake shows an increasing trend, while milk production remarkably increased after the SP supplement (*P* < 0.05). FCM also shows a significant increase after receiving SP treatment (*P* < 0.05). Then, the milk quality was measured, and the results indicate that no significant alterations were observed except the significant increase in milk fat content (*P* < 0.05).

Milk fat was further selected for the composition measurement after the SP supplement treatment, and the results are shown in Table 3. Milk fat composition mainly comprised SFA, which contributed to 65–70% of total milk fat and efficiently decreased after the SP treatment; MUFA, which contributed to 20–25% of the total milk fat and marginally increased after the SP supplement; and PUFA, which contributed to <5% of the total milk fat, but provided critical benefits for human health, and shows a significant enhancement in SP treatment compared with CON (*P* < 0.05). Specifically, SFAs such as capric acid and palmitic acid; MUFAs including oleic acid and methyl cis-10-pentadecenoate; and PUFAs involving linoleic acid, α-linolenic acid, and docosahexaenoic acid remarkably increased, while methyl hexanoate, dodecane lauric acid, octadecanoic acid, and elaidic acid significantly decreased after the SP supplement.

**Table 3:** Effects of small peptide supplement on milk fat compositions (%).

**Items**		**Name**	**SP (*n* = 8)**	**CON (*n* = 8)**	**SE**	***P*-value**
SFA	C4:0	Butyric acid	3.18	3.11	0.064	0.699
	C6:0	Methyl hexanoate	2.72	3.08	0.06	0.034
	C8:0	Methyl octanoate	1.37	1.28	0.082	0.106
	C10:0	Capric acid	3.02	2.81	0.088	0.038
	C11:0	Methyl undecanoate	0.069	0.04	0.010	0.175
	C12:0	Dodecane lauric acid	2.64	3.58	0.179	0.001
	C13:0	Methyl tridecanote	0.11	0.1	0.006	0.295
	C14:0	Myristoic acid	7.97	8.65	0.368	0.546
	C15:0	Methyl pentadecanoate	0.09	0.09	0.030	0.427
	C16:0	Palmitic acid	37.3	35.2	0.65	0.001
	C17:0	Methyl heptadecanoate	0.43	0.47	0.007	0.540
	C18:0	Octadecanoic acid	7.45	9.17	0.421	0.002
	C20:0	Eicosanoic acid	0.068	0.095	0.005	0.273
	C21:0	Methylheneicosanoate	0.33	0.42	0.015	0.101
	C22:0	Behenic acid	0.13	0.17	0.013	0.031
		Total	66.9	68.1	1.371	0.086
MUFA	C14:1	Methyl myristoleate	1.29	1.24	0.056	0.410
	C15:1	Methyl cis-10-pentadecenoate	0.32	0.27	0.009	0.039
	C16:1	Palmitoleic acid	1.57	1.63	0.065	0.231
	C17:1	Methyl cis-10-heptadecenoate	0.15	0.18	0.004	0.071
	C18:1	trans-9-octadecanoic acid (elaidic acid)	0.48	0.53	0.012	0.043
	C18:1	cis-9-octadecanoic acid (oleicacid)	19.8	18.9	0.66	0.033
	C20:1	Cis-11-eicosenoic acid	0.32	0.39	0.012	0.098
	C22:1	Cis-13- decosahedaenoic acid	0.15	0.19	0.01	0.064
		Total	24.1	23.3	0.64	0.066
PUFA	C18:2	translinoleic acid	0.073	0.086	0.007	0.407
	C18:2	linoleic acid	3.12	2.75	0.046	0.011
	C18:3 n6	Cis, cis, cis-6,9,12-octadecatrienoic acid (γ-linolenic acid)	0.32	0.45	0.044	0.162
	C18:3 n3	Cis, cis, cis-9,12,15-octadecatrienoic acid (α-linolenic acid)	0.39	0.34	0.017	0.031
	C20:2	Cis, cis, 11, 14-eicosadienoic acid	0.071	0.078	0.006	0.076
	C20:3 n3	Cis-11, 14, 17-eicosapentaenoic acid	0.09	0.12	0.005	0.054
	C20:4 n6	Arachidonic acid	0.11	0.13	0.008	0.089
	C22:6 n3	docosahexaenoic acid	0.23	0.14	0.013	0.006
	C22:5 n3	docosapentaenoic acid	0.022	0.016	0.007	0.067
		Total	4.35	4.08	0.116	0.013
Others			4.82	4.35	0.023	0.076

SP, Small peptide supplement treatment; CON, control treatment. SE, standard error; SFA, saturated fatty acid; MUFA, monounsaturated fatty acid; PUFA, polyunsaturated fatty acids.

### Ruminal volatile fatty acids measurement

Ruminal VFAs, especially the acetate, provided the primary substrates for milk fat synthesis. Therefore, rumen VFAs were measured, and the results are shown in Table 4. Total VFA accumulated significantly in the SP treatment. The rumen contents of acetate, isobutyrate, and butyrate were all significantly increased after the SP supplement (*P* < 0.05) compared with CON. Despite of insignificant increase, propionate content also performed an increasing trend after the SP treatment. Thus, no changes were observed in the acetate /propionate ratio because of the corporate enhancement of both acetate and propionate.

**Table 4:** Effects of dietary supplementation of small peptides on rumen fermentation.

**Items**	**SP (*n* = 8)**	**CON (*n* = 8)**	**SE**	***P*-value**
Ruminal pH	6.08	6.14	0.08	0.351
Acetate (mmol/L)	67.67	59.29	3.97	0.047
Propionate (mmol/L)	23.79	20.44	1.65	0.054
Isobutyrate (mmol/L)	1.17	0.65	0.16	0.004
Butyrate (mmol/L)	16.52	14.25	1.08	0.049
Valetate (mmol/L)	1.95	2.07	0.37	0.744
Isovaletate (mmol/L)	2.40	2.44	0.34	0.915
TVFA (mmol/L)	113.51	99.15	6.88	0.035
A/P	2.88	2.92	0.09	0.622

SP, Small peptide supplement treatment; CON, control treatment; SE, standard error; A/P, acetate to propionate ratio; TVFA, total volatile fatty acid.

### Rumen lipometabolic-related metabolites measurement

A total of 1,160 metabolites were identified through the LC-MS measurement methods across all samples by retaining the treatments with a null value of ≤ 50%. All identified metabolites were provided in Supplementary Table 2. The metabolites were then identified and clustered into different functional categories based on their characters. Those metabolites, which clustered into lipid metabolism, were then selected for the following differential and functional analyses.

**Figure 1:**
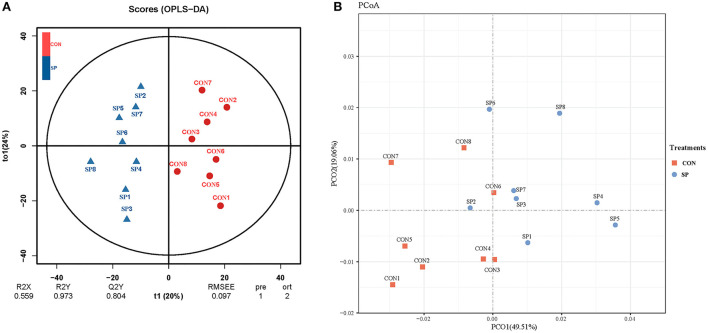
Principal components **(A)** and orthogonal partial least squares discriminant analysis (OPLS-DA) plot **(B)** analysis on the ruminal lipometabolic-related metabolites between small peptide supplement and control treatments. CON, control treatment, SP, small peptide treatment.

Differential analysis of the lipometabolic-related metabolites between SP and CON was first conducted through PCA and OPLS-DA analysis. As shown in Figure 1A, PC1 and PC2 accounted for 45.6 and 22.6% of the total variation, respectively. Lipometabolic-related metabolites showed a significant alteration after the SP treatment. The samples of SP treatment could clearly separate from that of CON, in both PCA and OPLS-DA analysis, as shown in Figure 1B.

Significantly altered lipometabolic metabolites under SP treatment compared with CON were analyzed based on the statistical standard of fold change >2, VIP>1, and *P* < 0.05, and all the results are shown in Table 5. A total of 35 significant differential altered metabolites including 27 upregulated and eight downregulated metabolites were detected based on the filtering standard. The upregulated metabolites mainly consist of phosphatidylcholine (PC), phosphatidylethanolamine (PE), phosphatidylinositol (PI), phosphatidylserine (PS), triacylglycerol (TG), and Acetyl-CoA. Meanwhile, the downregulated metabolites cluster into diacylglycerol (DG) and phosphatidic acid (PA), which mainly consist of saturated fatty acids.

**Table 5:** Significantly changed rumen lipometabolic-related metabolites between small peptide supplement treatment and the control treatment.

**Items**	**log_2_ FC**	***P*-value**	**VIP**
Acetyl-CoA	1.34	0.025	1.34
Biotin	1.43	0.039	1.04
Biotinyl-5'-AMP	1.44	0.046	1.24
PI(18:1(9Z)/0:0)	1.71	0.011	1.68
PI(12:0/0:0)	1.74	0.021	1.57
PE(18:1(11Z)/18:1(11Z))	1.80	0.012	1.67
PC(18:1(9Z)/0:0)	1.84	0.015	1.70
PI(16:1(9Z)/16:1(9Z))	1.91	0.017	1.72
PE(18:0/18:1(9Z))	2.15	0.042	1.42
PC(14:0/0:0)	2.21	0.002	1.74
LysoPC(18:1(11Z))	2.34	0.027	1.56
PE(18:0/0:0)	2.45	0.038	1.43
PC(16:1(9Z)/0:0)	2.46	0.005	1.83
PC(16:0/22:6(4Z,7Z,10Z,13Z,16Z,19Z))	2.49	0.010	1.66
PC(18:1(9Z)/20:5(5Z,8Z,11Z,14Z,17Z))	2.54	0.018	1.66
PC(20:2(11Z,14Z)/P-18:1(11Z))	2.71	0.024	1.69
PS(17:0/19:0)	2.85	0.042	1.39
PS(19:0/19:0)	2.89	0.038	1.23
PS(18:1(9Z)/0:0)	3.22	0.013	1.63
LysoPC(20:4(5Z,8Z,11Z,14Z))	3.55	0.031	1.53
PS(18:1(11Z)/18:1(11Z))	3.72	0.104	1.21
PC(14:0/P-18:0)	3.74	0.022	1.55
PC(o-16:1(9Z)/22:0)	3.80	0.014	1.72
TG(14:1(9Z)/16:1(9Z)/16:1(9Z))	4.18	0.023	1.55
PC(16:0/22:5(7Z,10Z,13Z,16Z,19Z))	4.39	0.029	1.23
CDP-DG(18:1(11Z)/16:0)	4.50	0.045	1.38
IPC 18:0;3/24:0;2	5.87	0.017	1.85
PS(16:0/16:0)	5.47	0.032	1.52
Stearic acid	−3.84	0.035	1.47
Nicotinic acid	−1.36	0.05	1.56
PA(14:0/0:0)	−3.04	0.019	1.06
TG(16:0/16:1(9Z)/18:1(9Z))	−2.46	0.029	1.20
PE(14:0/14:0)	−2.26	0.021	1.74
PG(16:0/18:1(11Z))	−2.23	0.022	1.48
DG(14:0/16:1(9Z)/0:0)	−2.12	0.028	1.62

SP, Small peptide supplement treatment; FC, fold change; CON, control treatment; VIP, Variable importance in the projection; PA, phosphatidic acid; PC, phosphatidylcholine; PE, phosphatidylethanolamine; PG, phosphatidylglycerol; PI, phosphatidylinositol; PS, phosphatidylserine; TG, triacylglycerol; DG, diacylglycerol.

**Figure 2:**
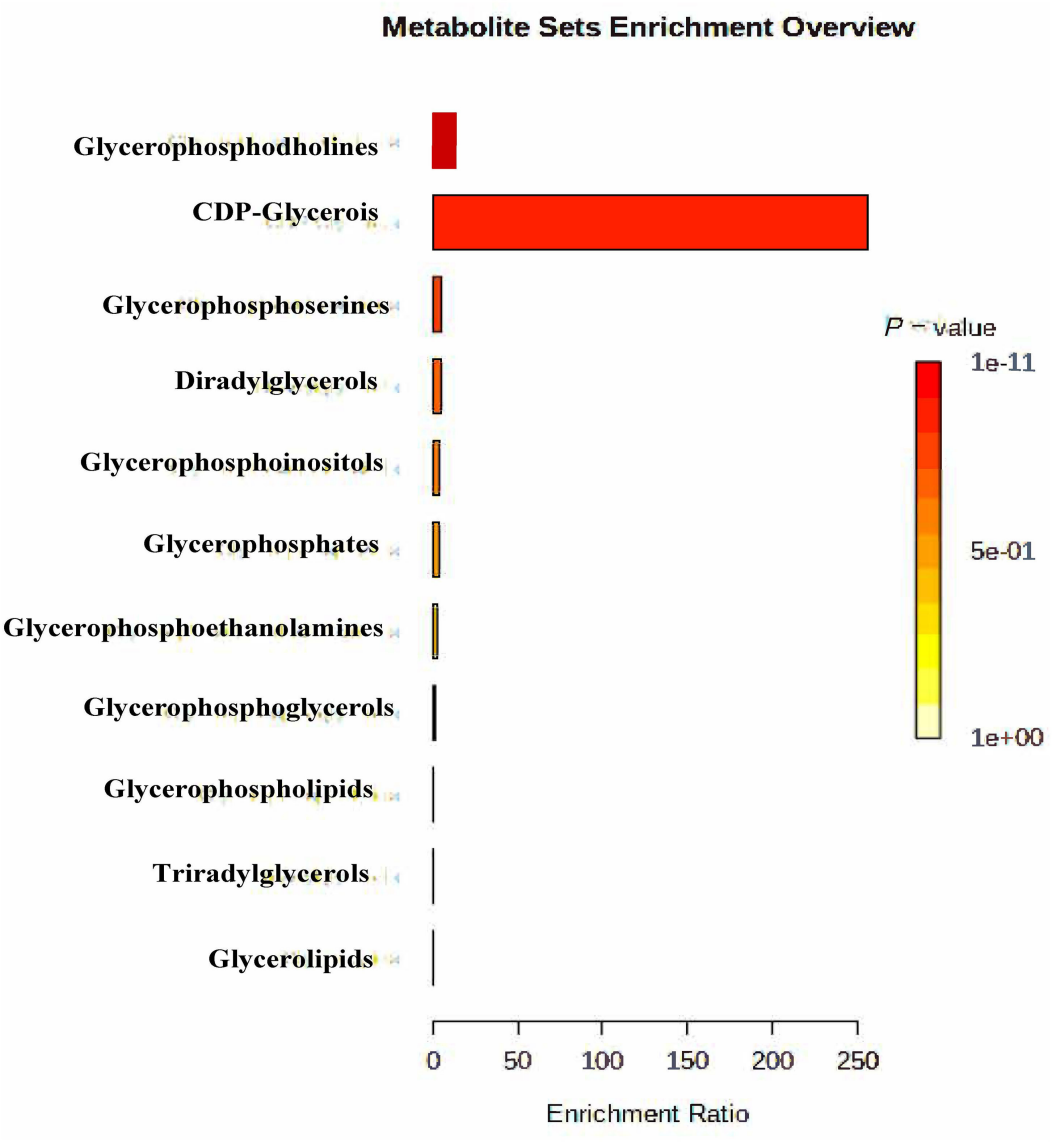
Enrichment functional analysis on the differential rumen lipometabolic-related metabolites between SP and CON treatments. CON, control treatment; SP, small peptide treatment.

At last, the functional enrichment analysis was applied based on all differential metabolites to determine the mainly altered functions. All enrichment results are shown in Figure 2.

Figure 2 shows the critical lipometabolic functions altered between SP supplement and control treatments. The most significant altered functions are glycerophosphocholines, followed by glycerophosphoserines. In addition, glycerolipids and glycerophosphoinositlols were also detected with significant enrichment between SP and CON treatment. All these alterations were part of the lipometabolic pathways and may further impact lipid metabolism synergistically.

### Ruminal microbial communities measurement

Rumen microbial communities were further measured to investigate the underlying mechanism of altered rumen metabolism under SP supplement treatment. In general, a total of 4,523 OTUs were obtained by performing OTU clustering on non-repetitive sequences according to 97% similarity. Then, 15 phyla and 2,534 genera were identified after quality control, and all the taxonomic information is displayed in Supplementary Table 3. All taxonomic bacteria were applied for α-diversity, β-diversity, and differential community investigation.

#### α-diversity

Alpha diversity was first investigated to determine the complexity of rumen microbial diversity through Chao1, Shannon, Simpson, and ACE indexes, and all results are shown in Table 6. Alpha-diversity index of ACE increased significantly after the SP treatment (*P* < 0.05). Meanwhile, the other indexes increased after the SP supplement, however, not significantly.

**Table 6:** Effects of small peptides supplement on α-diversity of ruminal microbiota.

**Items**		**SP (*n* = 8)**	**CON (*n* = 8)**	**SE**	***P-value***
Shannon		8.26	8.16	0.15	0.182
Simpson		0.98	0.98	0.01	0.342
ACE		2,256.5	2,203.4	22.4	0.018
Chao1		2,156.3	2,131.4	31.2	0.062

SE, standard error of the mean; CON, control treatment; SP, small peptide treatment.

#### β-diversity

**Figure 3:**
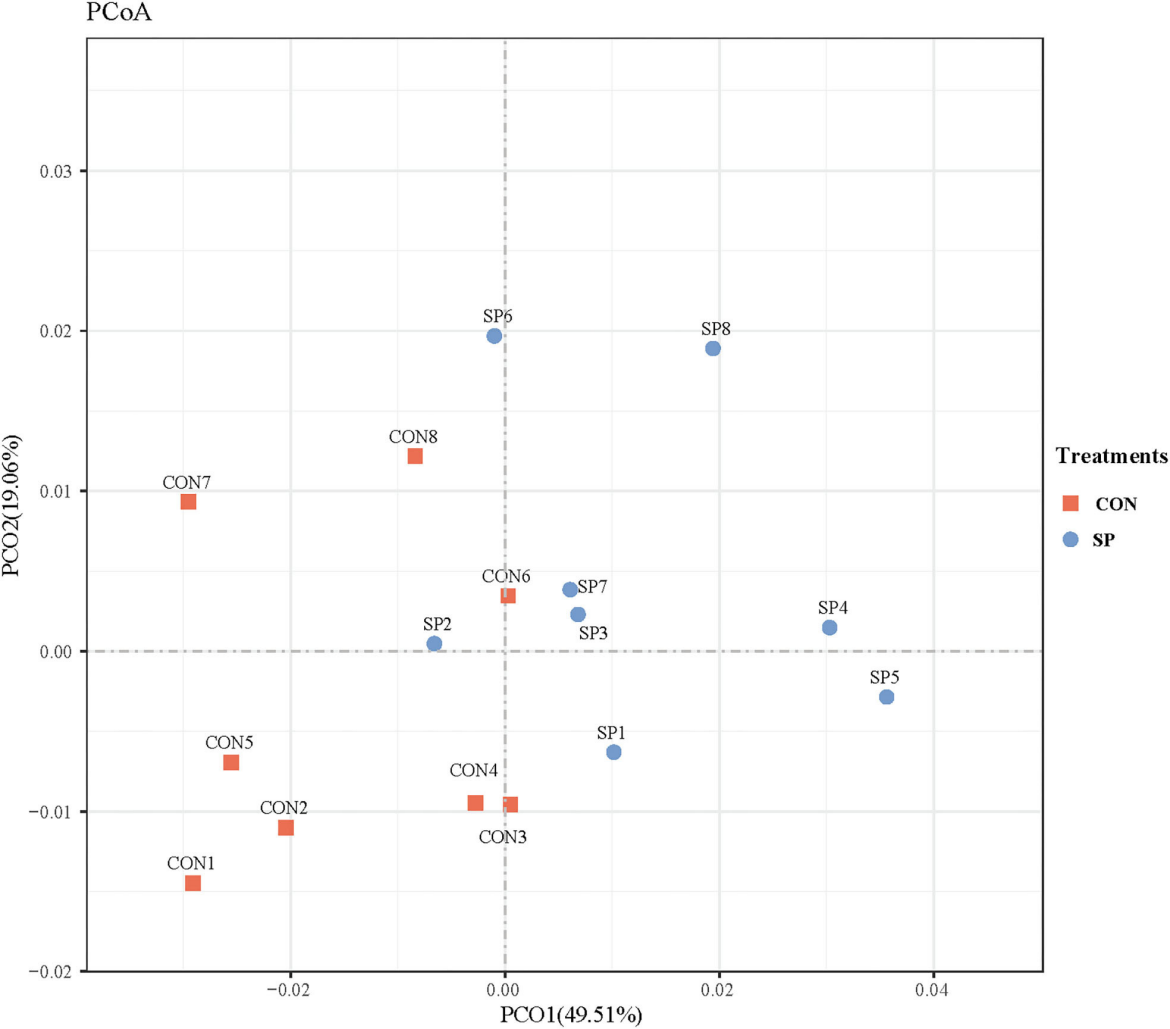
Principal coordinate analysis (PCoA) on community structures of the rumen microbiota between control treatment and small peptide supplement treatment. CON, control treatment; SP, small peptide treatment.

Differential analysis on rumen microbial communities between SP and CON was primarily proceeded through PCoA to investigate the alteration of whole communities. As shown in Figure 3, PCoA axes one and two accounted for 49.51 and 19.06%, respectively. Bacterial communities in SP treatment could be significantly separated from those in CON through PCoA axes one and two, except SP2.

Furthermore, differential analysis was applied based on the relative abundance of the bacterial community, and all the results are shown in Table 7. *Prevotella, Ruminococcaceae*, and *Succiniclasticum* contributed the most five abundant genera and accounted for nearly half of all microbiota profiles.

**Table 7:** Effects of small peptide supplement on relative abundances of ruminal bacteria communities (%).

**Items**	**SP (*n* = 8)**	**CON (*n* = 8)**	**SE**	***P*-value**
*g__Prevotella*	20.32	16.34	2.39	0.064
*g__Ruminococcaceae*	11.84	15.70	1.89	0.005
*g__Succiniclasticum*	10.89	9.51	0.82	0.291
*g__Lachnospiraceae*	5.22	5.28	0.28	0.623
*g__Eubacterium*	4.84	3.97	0.21	0.141
*g__Rikenellaceae*	1.77	3.14	0.25	0.015
*g__Ruminococcus*	3.77	7.31	1.88	0.012
*g__Shuttleworthia*	2.72	1.34	0.52	0.032
*g__Prevotellaceae*	1.27	1.22	0.23	0.371
*g__Acetitomaculum*	1.28	0.73	0.33	0.023
*g__Erysipelotrichaceae*	1.11	0.86	0.33	0.137
*g__Lachnoclostridium*	0.66	0.73	0.22	0.995
*g__Saccharofermentans*	0.63	0.45	0.11	0.008
*g__Butyrivibrio*	0.48	0.40	0.05	0.048
*g__Ruminiclostridium*	0.21	0.28	0.08	0.503
*g__Lachnospira*	0.32	0.16	0.07	0.034
*g__Pseudobutyrivibrio*	0.11	0.18	0.03	0.009
*g__Acidaminococcus*	0.13	0.07	0.07	0.167
*g__Selenomonas*	0.04	0.11	0.04	0.001
*g__Lactobacillus*	0.05	0.06	0.05	0.936
*g__Pseudoramibacter*	0.04	0.02	0.03	0.089
*g__Bifidobacterium*	0.05	0.03	0.01	0.027
*g__Escherichia-Shigella*	0.01	0.01	0.003	0.68
*g__Bacteroides*	0.01	0.02	0.01	0.571
*g__Succinivibrio*	0.03	0.01	0.003	0.001
*g__Streptococcus*	0.01	0.01	0.006	0.083
*g__Butyricicoccus*	0.01	0.01	0.007	0.969
Others	31.63	30.72	1.45	0.211

SP, Small peptide supplement treatment; CON, control treatment; SE, standard error.

Microbial communities including *Shuttleworthia, Succiniclasticum, Bifidobacterium, Saccharofermentans, Acetitomaculum*, and *Succinivibrio* significantly proliferated, while the growth of *Ruminococcaceae, Pseudobutyrivibrio*, and *Selenomonas* significantly suppressed after the SP supplement. No significant changes were found for other bacterial genera.

### Interactive analysis between bacteria and lipometabolic-related parameters

**Figure 4:**
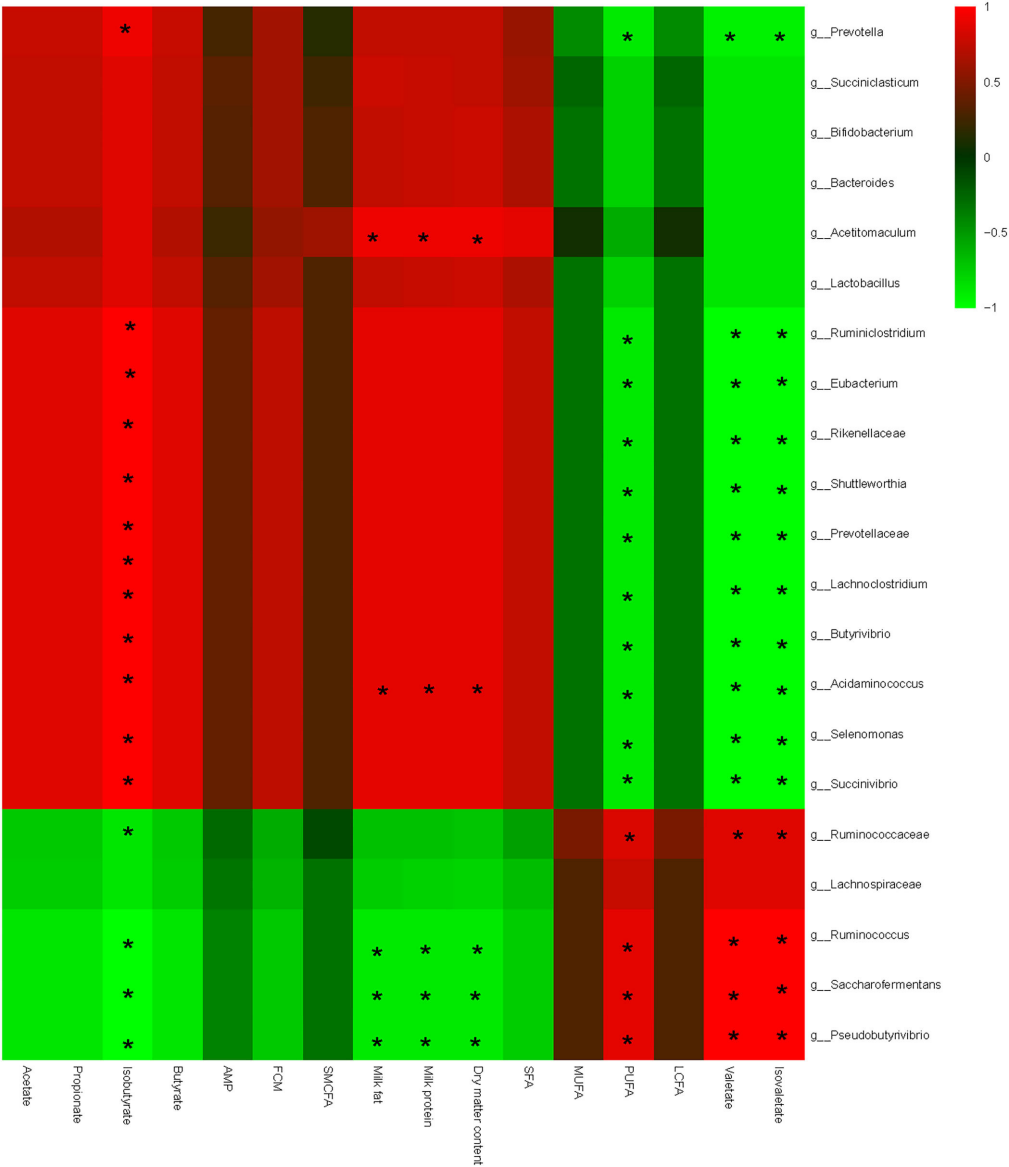
Correlation analyses between relative abundances of ruminal bacteria and ruminal fermentation parameters, milk quality, and milk fat composition on the level of genera. The red color represents a positive correlation, while the green color represents a negative correlation. “*” means a significant correlation (|*r*| > 0.55, *P* < 0.05).

Interactive analysis between the most abundant bacteria and lipometabolic-related parameters was ultimately conducted, and the result is shown in Figure 4.

In addition, bacteria could be clearly separated into two big clusters based on the relationship with lipometabolic-related parameters. One cluster mainly consists of *Shuttleworthia, Succiniclasticum, Bifidobacterium, Saccharofermentans, Lactobacillus, Acetitomaculum*, and *Succinivibrio*; and shows a negative correlation with PUFA, valerate, and isovalerate, and a positive correlation with acetate, butyrate, milk fat, and milk protein content. The other cluster mainly consists of *Ruminococcaceae, Pseudobutyrivibrio*, and *Saccharofermentans*, which shows a converse correlation with lipometabolic-related parameters compared with the prior one. Specifically speaking, milk fat content shows a significant negative correlation with *Pseudobutyrivibrio, Ruminococcus*, and *Saccharofermentans*, and a positive correlation with *Acetitomaculum* and *Acidaminococcus*. Likewise, the milk protein content shows a similar correlation compared with the milk fat. Conversely, PUFA, valerate, and isovalerate present a contrary correlation compared with the milk fat; milk protein shows a positive correlation with *Ruminococcaceae, Pseudobutyrivibrio, Ruminococcus*, and *Saccharofermentans*, and a negative correlation with *Shuttleworthia, Succiniclasticum, Bifidobacterium, Saccharofermentans, Lactobacillus, Acetitomaculum*, and *Succinivibrio*. Noteworthy considering that, isobutyrate shows a complete inverse correlation with the bacterial communities compared with valerate and isovalerate. No significant correlations were found among other parameters.

## Discussion

As one of the major assessment indices of milk quality, milk fat provided the essential FA for human beings. Milk fat content is mainly derived from blood transportation of the integrated FAs, which were synthesized in the rumen or other organs, or *de novo* synthesis in the mammary gland that required acetate generated from rumen degradation. Thus, in the following, the underlying mechanism of SPs on lipid metabolism would be discussed through the aspects of regulatory effects on substrates generated for mammary gland FA synthesis and effects on rumen lipometabolic efficiency.

### Effects of small peptide supplement on production performances

In this study, production performances, including DMI, milk yield, and milk fat content, and VFAs, including acetate and butyrate, significantly increased after the SP supplement. The main causative factor that helped in increasing fermentable parameters and production performances should be attributed to the proliferation of rumen microbiota after the SP supplement. In ruminal conditions, the rumen microbiome utilized carbohydrates and nitrogen for autologous proliferation (23). RDP provided the essential demand for nitrogen sources and further promoted the proliferation of rumen communities. As an easily degradable and utilized nitrogen source, the supplement of SPs supplies more degradable nitrogen for microbial proliferation, and the enhanced α-diversity may verify the proliferation of rumen communities and thereby increase rumen fermentation to provide more VFAs (24, 25). Besides, VFAs supply the most energy for physiological activities including exercises and lactation. Milk quality parameters such as milk fat rely largely on the provision of VFAs. The increased total VFAs after the SP supplement contributed to the promoted production performances.

### Effects of SP supplement on milk fat content

Milk fat in this study significantly increased after the SP supplement. Reasons may attribute to both the fatty acid synthesis process and the rumen lipometabolic process. Mammary *de novo* synthesis required the substances of acetate and butyrate which are mainly generated in rumen fermentation by the numerous quantities of rumen microbiome and transported through blood circulation providing the primary resources of milk fat (26). Therefore, increasing the rumen microbiota helps in the generation of acetate. Acetate-producing bacteria such as *Acetitomaculum, Bifidobacterium*, and *Succinivibrio* (27, 28) significantly increased after the SP supplement, which may further contribute to the enhancement of acetate content.

Moreover, acetate is a significant positive regulator in the milk fat synthesis process (5) and promotes the activation of lipid synthesis enzymes including acetyl-CoA carboxylase (ACACA), fatty acid synthase (FASN), and acyl-CoA synthetase short-chain family member 2 (ACSS2) (29–31). The increasing substrates for the enzymatic synthesis process catalyzed the synthesis reaction and then further improved the milk fat content under SP supplement treatment. Besides, the synthesis of FAs is an energy-consuming process that needs more ATP to catalyze the synthetic process. Interestingly, VFAs provided the main energy for both organism development and nutrient metabolism process (32) and also observed a significant increase after the SP treatment. The increased VFAs might generate more ATPs and provide more energy for milk fat synthesis, consequently increasing the milk fat content.

Apart from the *de novo* synthesis, another extreme source of milk fat content was the hydrolysis and absorption of dietary lipid content. On entering the rumen, dietary lipids were hydrolyzed by the lipases of five Gram-negative, curved rods, which had morphological and biochemical properties characteristic of the genus *Butyrivibrio* (33). In this study, *Butyrivibrio* showed a significant increase after the SP supplement, which hydrolyzed more lipids into FAs and transported them into the mammary gland to increase the milk fat content. Besides, FA biosynthesis was also demonstrated utilizing both branched-chain and straight-chain precursors, which were generated from amino acids in mixed rumen bacteria (34–36). Supplement of SPs may further degrade into branched-chain and straight-chain amino acids and provide more substrates for FA synthesis, and finally, more FAs were synthesized.

Moreover, the supplement of SPs fill the gap of deficient RDP requirements to satisfy the energy needs. Re-establishment of RENB considerably proliferated the cellulolytic bacteria such as *Acetitomaculum*, and *Bifidobacterium* (28), which were found to be significantly proliferated in this study, causatively induced the increase of acetate, offered more substrates for milk fat synthesis, and, therefore, contributed to the increase of milk fat content.

### Effects of SP supplement on polyunsaturated fatty acids

Conventionally, various plant lipids were hydrogenated prior to incorporation into bacterial membrane lipids by neither Group A bacteria including *Ruminococcus* spp. and *Lactobacillus* spp., which could hydrogenate linoleic acid (LA) or linolenic acid (LNA) to trans-vaccenic acid (TVA), nor group B bacteria such as *B. proteoclasticum*, which could complete all steps of biohydrogenation (BH). However, when vaccenic acid accumulates, the final BH step is inhibited (37–39). In this study, *Ruminococcus* significantly decreased in the SP treatment, combined with the accumulation of vaccenic acid detected by rumen fluid metabolomic measurement, may partially interpret the increase of PUFA.

In summary, the SP supplement helps for the proliferation of rumen bacterial communities, which further provides more substrates including acetate and butyrate for mammary milk fat synthesis. Besides, SP also promotes ruminal lipometabolism and therefore significantly increases the milk fat content.

## Data availability statement

The data presented in the study are deposited to NCBI SRA database, accession number BioProject ID: PRJNA867506.

## Ethics statement

The animal study was reviewed and approved by Animal Care and Use Committee of the Jiangxi Agricultural University.

## Author contributions

MQ and FX designed the study. EL, WX, and QP conducted the experiment. FX and WX contributed to the manuscript writing and English editing. EL, LX, LW, and KM contributed to the parameter measurement and the data analysis. All authors contributed to the article and approved the submitted version.

## Funding

This study was supported by the China Agriculture Research System of MOF and MARA (CARS-37), the Science and Technology Planning Project of Jiangxi Educational Department (GJJ200414), and the Latitudinal Project of Jiangxi Agricultural University (2021JXAUHX021).

## Conflict of interest

Authors WX and QP were employed by Chengdu Mytech Biotech Co., Ltd. Author WH was employed by Shanghai Menon Animal Nutrition Technology Co., Ltd. The remaining authors declare that the research was conducted in the absence of any commercial or financial relationships that could be construed as a potential conflict of interest.

## Publisher's note

All claims expressed in this article are solely those of the authors and do not necessarily represent those of their affiliated organizations, or those of the publisher, the editors and the reviewers. Any product that may be evaluated in this article, or claim that may be made by its manufacturer, is not guaranteed or endorsed by the publisher.

## Supplementary material

Supplementary Table 1 Milk production record of each cow.

Supplementary Table 2 Rumen metabolomic results between SP and CON treatments.

Supplementary Table 3 Taxonomy results of both SP and CON treatments of each sample.
